# Role of Oxidative Stress in the Genesis of Ventricular Arrhythmias

**DOI:** 10.3390/ijms21124200

**Published:** 2020-06-12

**Authors:** Adriana Adameova, Anureet K. Shah, Naranjan S. Dhalla

**Affiliations:** 1 Department of Pharmacology and Toxicology, Faculty of Pharmacy, Comenius University in Bratislava, and Center of Experimental Medicine, Slovak Academy of Sciences, Institute for Heart Research, Odbojarov 10, 83232 Bratislava, Slovakia; 2 Department of Kinesiology, Nutrition and Food Science, California State University, Los Angeles, CA 90032, USA; akaur23@calstatela.edu; 3 Institute of Cardiovascular Sciences, St. Boniface Hospital Albrechtsen Research Centre, and Department of Physiology & Pathophysiology, Max Rady College of Medicine, University of Manitoba, Winnipeg, MB R3E 0W2, Canada; nsdhalla@sbrc.ca

**Keywords:** ventricular arrhythmias, catecholamines, angiotensin II, ischemia-reperfusion injury, myocardial infarction, sudden cardiac death, antioxidant therapy

## Abstract

Ventricular arrhythmias, mainly lethal arrhythmias, such as ventricular tachycardia and fibrillation, may lead to sudden cardiac death. These are triggered as a result of cardiac injury due to chronic ischemia, acute myocardial infarction and various stressful conditions associated with increased levels of circulating catecholamines and angiotensin II. Several mechanisms have been proposed to underlie electrical instability of the heart promoting ventricular arrhythmias; however, oxidative stress which adversely affects ion homeostasis due to changes in the ion channel structure and function, seems to play a critical role in eliciting different types of ventricular arrhythmias. Prevention or mitigation of the severity of ventricular arrhythmias due to antioxidants has been indicated as the fundamental contribution in the field of preventive cardiology; however, novel interventions have to be developed for greater effectiveness and specificity in attenuating the adverse effects of oxidative stress. In this review, we have attempted to discuss proarrhythmic effects of oxidative stress differing in time and concentration dependence and highlight a molecular and cellular concept how it alters cardiac cell automaticity and conduction velocity sensitizing the probability of ventricular arrhythmias with resultant sudden cardiac death due to ischemic heart disease and other stressful situations. It is concluded that pharmacological approaches targeting multiple mechanisms besides oxidative stress might be more effective in the treatment of ventricular arrhythmias than current antiarrhythmic therapy.

## 1. Introduction

It was well known that a varying degree of atrial and ventricular arrhythmias occur in different types of heart diseases including heart failure, myocardial infarction, atherosclerosis, thrombosis, ischemia/reperfusion injury, diabetes and hypertension [1,2,3,4]. In spite of the fact that the pathogenesis and therapy of arrhythmias are complex problems, enormous advancement in the understanding of mechanisms of arrhythmias as well as progress in the development of antiarrhythmic strategies have been made in recent years [1,2,3,4]. Some arrhythmias are completely reversible while in many cases restoration and maintenance of sinus rhythm is poor. Such unsatisfactory results are considered to be caused by various factors, such as polymorbidity of patients, inter- and intra-individual variability in response to antiarrhythmic approaches as well as proarrhythmic potential of some antiarrhythmic therapies. Moreover, a diverse character of proarrhythmic substrates localized in the heart or in the circulatory system significantly determines the outcome and prognosis of myocardial injury as well as therapeutic efficacy of strategies used for the treatment of arrhythmias. It may be noted that antiarrhythmic drugs, commonly categorized according to the Vaughan Williams classification, have been designed to alter cardiac cell abnormal automaticity, excitability and conduction velocity through direct inhibition of the sodium, calcium and potassium channels, or indirectly through the inhibition of particular receptors coupled to these ion channels. Thus, by considering their mode of action and the fact that these modulate abnormalities in the ion currents, as well as the occurrence of re-entry and focal activities, it is assumed that antiarrhythmic drugs target downstream events/molecules of cardiac damage producing electrical instability rather than acting on the primary mechanisms for arrhythmias. Among several proposed mechanisms such as genetic alterations in ion channels, inflammation, and changes in myocardial metabolism, oxidative stress is considered as a key candidate for the fundamental mode as well as arrhythmias-promoting event. This article is therefore intended to focus mainly on the discussion regarding oxidative stress as the major mechanism for the development of cardiac arrhythmias.

The occurrence of oxidative stress in the heart has been considered to be a consequence of imbalances between the activities of pro-oxidant offensive and antioxidant defensive systems [5,6]. Indeed, various indicators of altered redox status including the levels of free oxygen radicals, levels of advanced glycation, lipid peroxidation end-products, as well as activities and levels of genes encoding particular enzymes have been found in the heart and blood of subjects with arrhythmias. Such changes indicating oxidative stress have been linked with atrial fibrillation [7,8], arrhythmias occurring in failing hearts [9,10,11,12] and rhythm disturbances in acute myocardial ischemia/reperfusion injury [13,14]. Likewise, other conditions being related to oxidative stress and ventricular arrhythmias include high levels of catecholamines [15,16,17], homocysteine [18,19] or iron overload [20]; arrhythmic changes under these situations are comparable to those observed upon treatment of the hearts with H_2_O_2_ [21], showing association with diminished electrical stability. These observations are consistent with the antiarrhythmic effects of different substances such as glutathione, superoxide dismutase, catalase, allopurinol and mito-tempo, which are known to react with reactive oxygen species (ROS) directly, and/or indirectly as inhibitors of the free radical generating enzymes, and/or activators of intracellular antioxidant enzymes [12,14,22,23,24]. On the other hand, there are some conflicting data showing failure of antioxidant interventions in the treatment of both ventricular and supraventricular arrhythmias. Accordingly, no antioxidant therapy has been recommended by the European Society of Cardiology and American Heart Association as a sole pharmacological approach to treat ventricular arrhythmias [1,3] or atrial fibrillation [2,4]. On the basis of such inconsistent data from both experimental and clinical studies, it is evident that there are several gaps for bridging oxidative stress with the genesis of arrhythmias. Since this phenomenon has attracted a great deal of attention over the past 3–4 decades, this review is an attempt to recapitulate the general aspects of deleterious effects of oxidative stress in arrhythmogenesis and to highlight its pivotal contribution to sudden cardiac death. In addition, it is planned to discuss a concept how oxidative stress, as an underlying cause of various cellular defects and molecular alterations, induces cardiac damage and ventricular arrhythmias in some cases of heart diseases.

## 2. Oxidative Stress as a Factor for Increasing the Risk of Arrhythmias

Effects of oxidative stress on heart function and morphology have been examined extensively and it is now widely accepted that it can induce events leading to various cellular defects upon releasing several inflammatory agents, altered membrane permeability and configuration of proteins [5,6]. These changes promote lipid peroxidation, protein oxidation, carbonylation, *N*-glycosylation, *S*-nitrosylation and other molecular abnormalities associated with altered ion homeostasis and higher production/release of certain molecules through leaky membranes with resultant functional modifications, including electrical instability of the heart [5,25,26,27]. Alterations in signaling due to oxidative stress have been found to adversely affect not only cardiomyocytes but also non-cardiomyocytes such as coronary artery smooth muscle cells [28,29,30], fibroblasts, [31] as well as infiltrating leucocytes [32], which themselves are able to generate ROS and thereby amplify deleterious environment. Thus, it is likely that the occurrence of myocardial damage, and severity of ventricular arrhythmias may significantly depend on a certain type of oxidative stress, post-translational changes in protein structure of cardiac cells as well as other associated cellular and molecular abnormalities. While acute ventricular arrhythmias are very likely triggered in response to rapid changes in oxidative status in the heart as seen with a rapid burst of ROS due to reperfusion of ischemic tissue, triggering of chronic ventricular arrhythmias might be continuously promoted due to sustained fluctuations in the cellular redox state as a consequence of long-term post-translational modifications of oxidative stress-related proteins as well as alterations in genotypes. Accordingly, this concept may also imply that efficacy/failure of a particular antioxidant strategy may be determined by a signaling model linking with particular cellular, molecular and functional abnormalities due to oxidative stress.

Ventricular arrhythmias involve both non-serious rhythm disorders such as premature ventricular beats, bigemines and trigemines, as well as life-threatening arrhythmias, such as ventricular tachycardia and fibrillation, which might lead to sudden cardiac death. These disturbances in cardiac rhythm are triggered by ischemia and their incidence increases rapidly by the restoration of the blood flow depending upon to the duration of ischemia [33,34,35]. Likewise, catecholamines overload, which may occur as a result of various stressful stimuli, ischemia, hypoxia and heart failure can induce changes in cardiac cells conduction velocity and automaticity resulting in ventricular arrhythmias [15,16]. It has been generally considered that ischemia and ischemia/reperfusion-induced ventricular arrhythmias occur as a direct stimulation of the beta-adrenergic receptors in the heart and increased production of cyclic AMP [33,36]. Pharmacological modulation of the beta-adrenergic receptors by propranolol, however, decreased ventricular fibrillation threshold only during ischemia but was without effect on the arrhythmias threshold during coronary artery release [33]. Likewise, other nonselective as well as selective beta-blockers have shown inconsistent beneficial effects on the ischemia/reperfusion-induced ventricular arrhythmias [33,37,38,39,40,41,42]. In fact, ventricular arrhythmias threshold, incidence and the overall severity due to the administration of excessive amount of catecholamines were not prevented by a beta-blockade [43]. Some of the results showing the ineffectiveness of a selective beta-adrenoceptors blocker, atenolol, in preventing various types of arrhythmias due to coronary artery occlusion [41] and catecholamines [43] are shown in Table 1 and Table 2, respectively. Since catecholamines-induced arrhythmias were also not modified by treatment with losartan (Table 2), it is evident that the toxic effects of catecholamines are not elicited through the release of angiotensin II [43]. Collectively, these data suggest that the beta-adrenoceptor associated mechanism is not exclusively involved in the genesis of ventricular arrhythmias, and that other factors acting either in its cooperation or solely might participate in the promotion of electrical instability under such conditions of ischemic heart disease.

Since high levels of ROS are generated during the development of ischemia/reperfusion injury as well as upon the oxidation of catecholamines by both enzymatic and non-enzymatic mechanism, it has been argued that ventricular arrhythmias are induced by oxidative stress in the myocardium [5,6,14,44,45,46,47,48]. This concept is consistent with observations [15] that treatment of animals with well-known antioxidants such as vitamin E and *N*-acetyl-cysteine prevented the epinephrine-induced different types of arrhythmias as well as increased the levels of plasma malondialdehyde, an index of oxidative stress (Table 3). Furthermore, different antioxidant vitamins such as vitamin A and vitamin C [16] as well as sulphur-containing amino acids, taurine and cysteine [26], have been reported to exert beneficial effects on catecholamines-induced arrhythmias. It may also be noted that several antioxidants have been observed to mitigate ischemia/reperfusion-induced ventricular arrhythmias and lipid peroxidation [49,50,51,52,53,54]. Although some of investigators have failed to show the beneficial effects of different antioxidants on cardiac rhythm disturbances due to ischemia/reperfusion induced injury [55,56,57,58], such conflicting results may be due to differences in the doses of antioxidants used in these studies or the experimental design. Nonetheless, treatment of animals with vitamin E was observed to attenuate different electrocardiographic abnormalities and lipid peroxidation at different times (1, 3, 7 and 21 days) of inducing myocardial infarction [59]. The data on electrocardiographic changes and indices of oxidative stress in the untreated and vitamin E-treated animals at 21 days after coronary artery occlusion are shown in Table 4 [59]. These results provide evidence to support the view that oxidative stress likely plays an important role in the development of electrical instability of the heart and genesis of ventricular arrhythmias.

In addition to increased levels of catecholamines due to the stimulation of the sympathetic nervous system, ischemic heart disease is known to be associated with high levels of circulating angiotensin II as a consequence of the activation of renin-angiotensin system [6,60]. The possibility of the involvement of angiotensin II in the genesis of ventricular arrhythmias was explored by treatment of ischemic hearts upon either angiotensin converting enzyme inhibition or angiotensin II receptor blockade. In this regard, several angiotensin converting enzyme inhibitors including captopril, ramipril and imidapril, which prevent the formation of angiotensin II, have been reported to reduce various types of arrhythmias due to ischemic heart disease [61,62,63,64,65]. The data for the electrocardiographic changes at 21 days after myocardial infarction [66] in the untreated and imidapril-treated animals are shown in Table 5. Furthermore, various angiotensin receptor blockers such as losartan have been shown to prevent the occurrence of arrhythmias due to ischemia/reperfusion injury [67,68,69,70,71]. It is pointed out that angiotensin II has been shown to produce ventricular arrhythmias [68,72] and activate NADPH oxidase to produce oxyradicals and subsequent oxidative stress [73,74]. Thus, the beneficial effects of angiotensin converting enzyme inhibitors as well as angiotensin II receptor blockers in attenuating ventricular arrhythmias in ischemic heart disease appears to be due to reduction in the degree of oxidative stress. Likewise, the prevention of ventricular arrhythmias due to coronary artery occlusion by a 5-hydroxytryptamine (5-HT) receptor inhibitor, sarpogrelate [17,75], may also be attributed to its effect on oxidative stress because the circulating level of serotonin (5-HT) was increased in myocardial infarction [76] and serotonin has been shown to generate oxyradicals upon oxidation due to monoamine oxidase [48].

## 3. Potential Mechanisms of Oxidative Stress Induced Ventricular Arrhythmias

Although proarrhythmic effects of oxidative stress are generally mediated due to abnormalities in cardiomyocyte Ca^2+^, K^+^ and Na^+^ homeostasis, as well as gap junction remodeling, alterations in sarcolemmal and sarcoplasmic reticulum (SR) Ca^2+^-handling proteins seem to play a critical role in producing defects in the automaticity, excitability and conductivity of cardiac cells. Modulation of sarcolemmal L-type Ca^2+^ channel, which is responsible for Ca^2+^ influx, via its α1C pore-forming subunit, by different oxidants has been shown to decrease the L-type Ca^2+^ current (*I*_Ca,L_); this effect is reversed by reducing the redox-sensitive groups [76,77]. The ryanodine receptor 2 (RyR2), the cardiac muscle specific isoform responsible for Ca^2+^-mediated Ca^2+^ release from SR, is also prone to oxidative stress. A number of studies have shown that ROS promote RyR sulfhydryl oxidation leading to reduced Ca^2+^ transients and enhanced SR Ca^2+^leak and thereby increase the risk for ventricular arrhythmias [78,79,80,81]. In this regard, it may be noted that genetic alterations in RyR2 are associated with catecholaminergic polymorphic ventricular tachycardia, a type of adrenergic-induced ventricular arrhythmias triggered by exercise and emotional stress, which may cause syncope and sudden cardiac death in the absence of any structural defect in the heart [82,83,84]. This condition interlinking oxidative damage due to catecholamines and Ca^2+^ dysregulation resulting in ventricular arrhythmias is in line with the concept developed in this review. In addition to these Ca^2+^-handling proteins, SR Ca^2+^-pump ATPase (SERCA), responsible for Ca^2+^ removal from the cytoplasm, also contains redox-sensitive residues, which might undergo oxidative changes. Oxidation of sulphydryl groups of SERCA impairs its activity indicating the occurrence of Ca^2+^ overload and concomitant action potential prolongation [85,86]. In contrast, pretreatment of ischemic/reperfused hearts with a mixture of catalase and SOD, the activities of which are decreased under such conditions [87], is capable of abolishing the abnormal SR Ca^2+^ uptake and preventing heart damage [88]. It is possible that oxidative stress may induce endoplasmic reticulum stress for causing ventricular arrhythmias; however, a detailed study in this regard needs to be carried out for establishing this mechanism. It is noteworthy that a sarcolemmal Ca^2+^-handling protein, Na^+^/Ca^2+^exchanger (NCX), associated with extrusion of the intracellular Ca^2+^, has also been shown to be stimulated by H_2_O_2_ [89], which can lead to the increased propensity for triggered arrhythmias due to delayed afterdepolarizations and arrhythmogenic transient inward current (*I*_ti_) [90,91].

**Figure 1:**
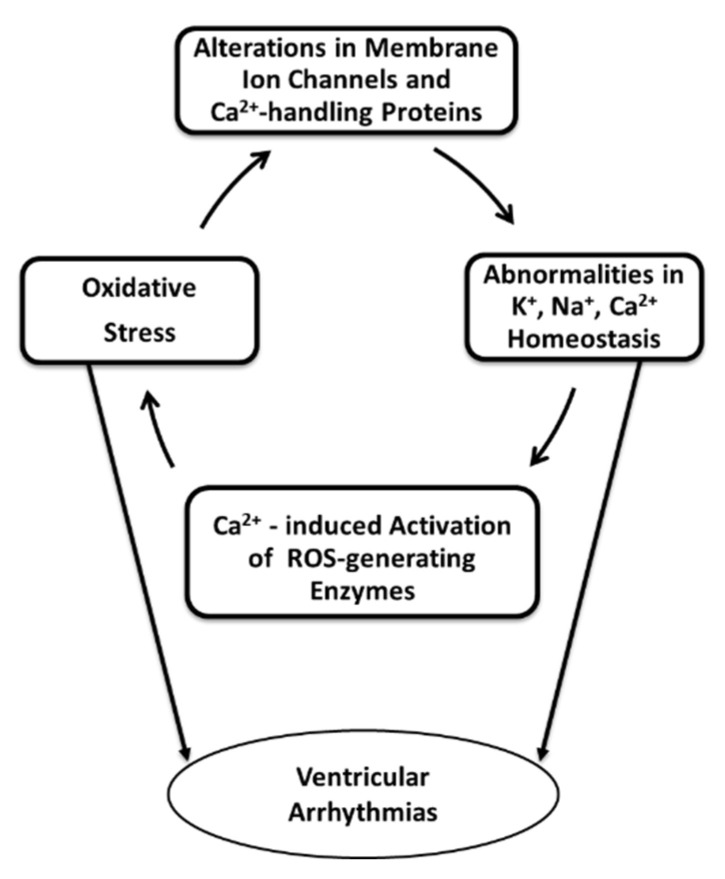
Stress-promoted alterations in ion homeostasis of cardiac cells inducing a further production of reactive oxygen species, thereby highlighting the probability of ventricular arrhythmias as a result of such viscous cycle.

In addition to changes in sarcolemmal and SR Ca^2+^-handling proteins due to oxidative stress, alterations in other Ca^2+^-related mechanisms may be involved in the genesis of ventricular arrhythmias. Oxidative activation has also been suggested to occur in Ca^2+^/calmodulin-dependent protein kinase II (CaMKII), a multifunctional protein kinase, which couples increases in cellular Ca^2+^ to fundamental responses in excitable cells [92]. Over-activation of this regulatory enzyme has been demonstrated to increase the arrhythmia risk due to an increase in early afterdepolarizations [93] and *I*_ti_ in the isolated rabbit ventricular myocyte stimulated with a prolonged action potential clamp [94]. In contrast, oxidized CaMKII was downregulated at the end of reperfusion compared to the levels before ischemia thereby arguing against its proarrhythmic potential during the reperfusion phase, although its over-activation through oxidation in earlier periods of the restoration of the blood cannot be ruled out [95]. Furthermore, such alterations in Ca^2+^ homeostasis resulting in Ca^2+^ overload and thereby the conversion of xanthine dehydrogenase to xanthine oxidase can further promote generation of superoxide radicals [27]. Elevation in the intracellular level of Ca^2+^ may also activate phospholipase C to stimulate arachidonic acid metabolism, which in turn generates oxygen free radicals as by-products [96]. Such Ca^2+^-induced reinforcement of ROS production indicating a viscous cycle for mediating cardiac damage and electrical instability is depicted in Figure 1.

Oxidative stress may also induce changes in Na^+^ and K^+^ homeostasis by affecting the sarcolemmal channels for these ions. H_2_O_2_ treatment has been found to affect K^+^ outward and inward rectifying channels. It accelerated *I*_Kr_ currents (through the channels encoded by human ether-a-go-go-related gene—hERG) and shortened the action potential duration. Likewise, H_2_O_2_ treatment induced faster hERG channel deactivation what might suggest the reduction of K^+^ conductance and increasing the risk of some ventricular arrhythmias during reperfusion [97]. High concentrations of NO increased inward rectifier *I*_K1_ and *I*_Kir_2.1 and the latter was accompanied by the increased channel opening probability due to S-nitrosylation of Cys76 residue in Kir2. On the other hand, such post-translational modification was found to be decreased as NO bioavailability was depressed in atrial fibrillation [98]. These findings may indicate that *S*-nitrosylation of Cys76 residue in K_ir_2 channel plays a major role in the modulation of action potential and higher risk for supraventricular arrhythmias. It should also be noted that altered transcriptional regulation of genes encoding ion channels has been suggested as other mechanisms of oxidative stress promoting ventricular arrhythmias. In fact, oxidative stress mediated by angiotensin II resulting in the production of H_2_O_2_, has been shown to reduce transcription of the cardiac SCN5A gene encoding Na^+^ channel. The corresponding decrease in mRNA abundance resulted in downregulation of cardiac Na^+^ channel current due to the binding of a transcriptional factor NF-κB to the respective SCN5A promoter [99]. Likewise, gap junction channels, made of connexions (Cxs), are also sensitive to ROS. A potential link of such negative interaction involves activation of the c-Src tyrosine kinase, which in turn has been shown to compete with Cxs and thereby inducing their downregulation and modification in the conductivity of electrical impulse [100,101]. Consistent with this proposed pathway are the observations that a specific inhibitor of c-Src, decreased the activation and levels of this kinase, upregulated Cxs levels, increased Cx43 at the gap junctions as well as their communication with resultant reduction of ventricular tachycardia inducibility [102].

It is noteworthy to point out that in addition to sarcolemmal and sarcoplasmic reticulum defects, changes in mitochondrial function are also considered to promote and amplify arrhythmogenesis. The exposure of mitochondria to oxidative stress has been shown to further result in a ROS production, an event called as ROS-induced ROS release (RIRR) [103,104]. The requisite threshold level of ROS can lead to the opening of mitochondrial permeability transition (MPT) pore and/or the inner membrane anion channel (IMAC) with resultant simultaneous collapse of mitochondrial inner membrane potential (Δψ) and transient increased ROS generation by the electron transfer chain [103,105]. The generated ROS are released in the cytosol and can further stimulate superoxide-mediated depolarization of neighboring mitochondria, thereby potentiating a massive ROS production and cellular damage, including the increased propensity for arrhythmias. The mitochondrial ROS-dependent oscillations in ΔΨm may lead to a decrease in ATP production and thus promote the cyclical activation of sarcolemmal KATP channels (sarcKATP channels) for increasing potassium conductance as well as shortening the effective refractory period (ERP) of the myocardium [106,107]. These alterations can be seen to induce rapid and heterogeneous action potential duration shortening and generation of potentially fatal arrhythmias [108,109]. Predisposition of the heart to such electrical dysfunction and generation of fatal arrhythmias is high under conditions referred to as metabolic sinks [110] with compromised energetic status due to hypoxia or ischemia. While these conditions and sarcKATP channel openers predispose the heart to arrhythmogenesis [106,107], the blockers of sarcKATP channels increase ERP [111] and prevent the occurrence of ventricular arrhythmias [112,113,114,115]. On the other hand, the opening of sarcKATP channels can be vital to cellular survival in the face of these conditions and in fact, the pharmacological blockade of sarcKATP channels was shown to increase cell death in hearts exposed to ischemia/reperfusion [116,117].

The role of mitochondrial RIRR in arrhythmogenesis has also been confirmed by some pharmacological studies. Treatment of the heart with a synthetic superoxide dismutase/catalase mimetic was found to abolish the H_2_O_2_-mediated release of O_2_^−^ and this was accompanied by the lower incidence of arrhythmias. Likewise, 4′-chlorodiazepam, an antagonist of a mitochondrial benzodiazepine receptor (mBzR), which modulates both IMAC and MPT [118,119], suppressed the incidence of the RIRR-induced ventricular arrhythmias by blunting the O_2_^−^ levels [105]. Other mBzR ligands (SSR180575 and PK11195) were also shown to eliminate typical electrophysiological responses to ischemia/reperfusion and oxidative stress [118,120]. Treatment with an agonist of the mBzR, recently renamed as the mitochondrial translocator protein (mitoTSPO) [119], exacerbated ischemia/reperfusion-induced electrical instability of the heart [110]. Thus, these observations indicate RIRR-mediated destabilizing effects on the action potential through modulating the IMAC and MPT and suggest that antioxidant therapy acting alone and/or in combination with inhibition the mitochondrial benzodiazepine receptor could alleviate abnormal electrical activation and reduce the risk of arrhythmias.

**Figure 2:**
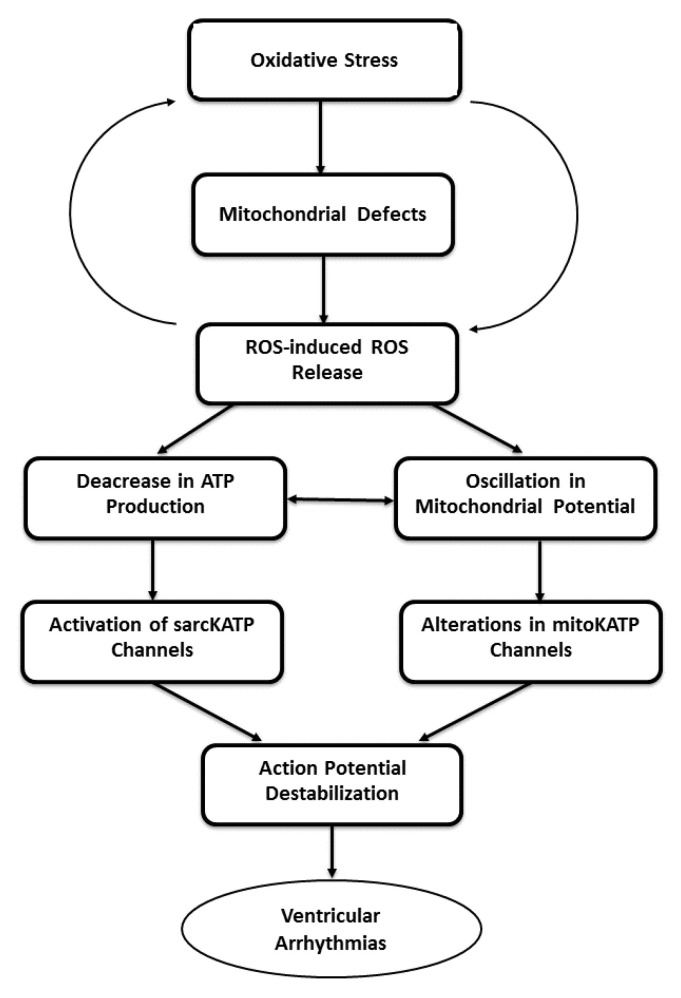
Events depicting the role of mitochondrial defects, reactive oxygen species (ROS)-released ROS and mitochondrial and sarcolemmal KATP channels in the generation of ventricular arrhythmias. ROS—reactive oxygen species; sarcKATP—sarcolemmal KATP channels and mitoKATP—mitochondrial KATP channels.

In contrast to the role of sarcKATP channels in arrhythmogenesis, the opening of mitochondrial ATP-sensitive potassium (mitoKATP) channels is cardioprotective. A mitoKATP opener (diazoxide) has been shown to reduce the incidence of ischemia/reperfusion-induced ventricular arrhythmias [121,122,123]. Likewise, mitoKATP opening has been indicated in some types of preconditioning, including ischemic preconditioning [121,122,124], pharmacological preconditioning mediated by noradrenaline [124], propofol [123], adenosine [125], opioids [126,127], nitroglycerine [128] and PPAR-alpha activation [13]. A temporal increase in ROS production in the preconditioning phase while attenuating ROS production during prolonged ischemia has been suggested as a potential mechanism of protection conferred by mitoKATP opening [122]. On the other hand, the massive ROS production activates sarcKATP for ensuring proarrhythmic effects. Collectively, these findings highlight that there is a fine line between ROS generation and action potential destabilization and that mitochondrial channels as well as other sarcolemma proteins regulating ion homeostasis are pivotal in the regulation of this process. A mechanistic link indicating a role of RIRR in mitochondrial defects promoting action potential destabilization with resultant triggering of ventricular arrhythmias is depicted in Figure 2.

## 4. Concluding Remarks

**Figure 3:**
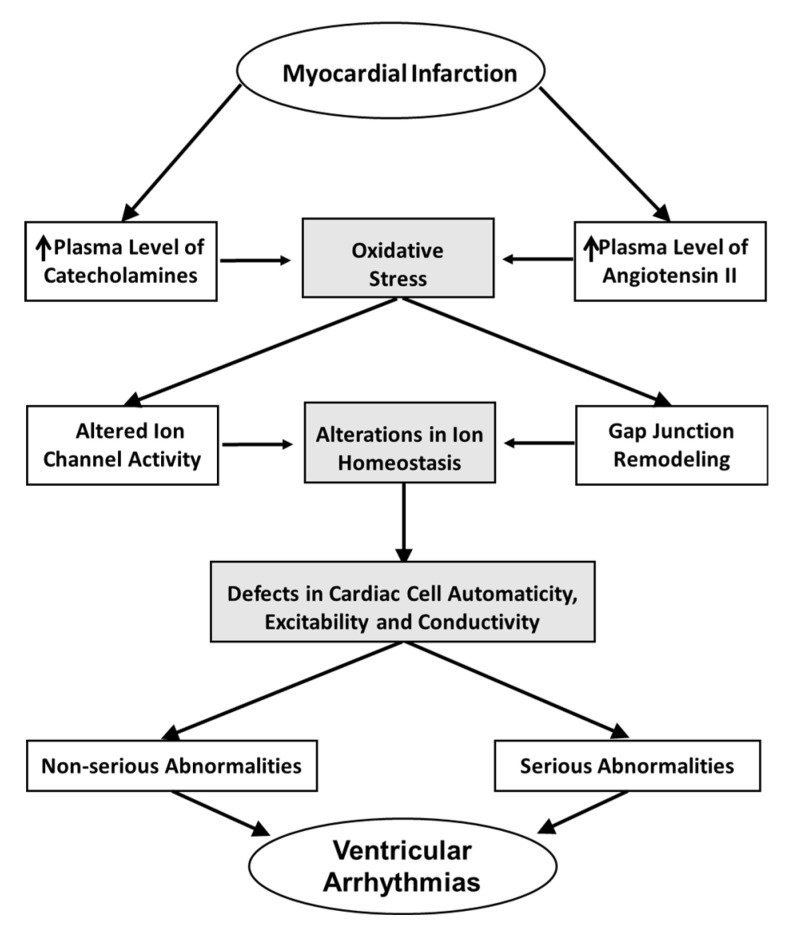
A scheme showing the role of high circulating levels of both angiotensin II and catecholamines in the generation of oxidative stress and subsequent events leading to the development of ventricular arrhythmias associated with acute myocardial infarction.

From the forgoing discussion it is apparent that oxidative stress plays a fundamental role in the genesis of ventricular arrhythmias under various conditions. It is also known that it can produce temporary or long-term changes promoting acute and chronic, non-serious or potentially lethal ventricular arrhythmias. Severe cellular and molecular abnormalities due to oxidative stress, occurring probably due to a rapid burst of ROS over-production or due to the modification of transcriptional processes, result in changes in the abundance of respective proteins and high probability of serious ventricular arrhythmias inducibility and sudden cardiac death. Notably, some mechanisms occurring in response to oxidative stress obviously overlap resulting in the reinforcement of altered redox cellular state and finally greater cellular damage as a consequence of such a viscous cycle. Thus, on the one hand, oxidative stress can be considered as an upstream signal to altered ion homeostasis, and gap junction remodeling indicating the promotion of ventricular arrhythmias. On the other hand, as oxidative stress can be reinforced by activating other ROS-generating enzymes due to changes in ion homeostasis of cardiac cells, it can be a downstream event making the cellular damage more complex. As a result, this paradigm may indicate the likelihood of ventricular arrhythmias of greater severity as well as for a long-term persistence. As experimental and clinical studies have shown inconsistent evidence of the efficacy of antioxidant approaches it is likely that other mechanisms such as inflammation and metabolic changes acting in cooperation with oxidative stress may contribute to the genesis of ventricular arrhythmias under certain conditions. Although from a pharmacotherapeutic perspective, it can be suggested that the modification of oxidative stress might serve as an effective tool capable of improving some mechanisms underlying the automaticity, excitability and conductivity of the heart with resultant attenuated arrhythmogenesis, an approach targeting multiple mechanisms may be necessary to prevent ventricular arrhythmias and sudden cardiac death. It seems that antioxidant therapy may be more beneficial from the prevention viewpoint rather than for the treatment of ventricular arrhythmias. Nonetheless, on the basis of information outlined in the article, a scheme depicting major events for the generation of oxidative stress as well as its consequences leading to the development of ventricular arrhythmias due to acute myocardial infarction is shown in Figure 3.

## Figures and Tables

**Table 1:** Incidence and duration of different types of arrhythmias due to coronary artery occlusion in rats with or without beta-adrenoceptor blocker, atenolol (20 mg/kg/day) treatment for 14 days.

Parameters	Incidence (%)		Duration (s)	
	Untreated	Atenolol-Treated	Untreated	Atenolol-Treated
Ventricular premature beats	100	100	1.98 ± 0.45	3.95 ± 1.44
Bigemines	73	77	5.23 ± 2.38	7.21 ± 2.11
Trigemines	ND	9	ND	0.19 ± 0.19
Salvos	63	65	0.54 ± 0.19	0.66 ± 0.25
Ventricular tachycardia	100	90	44.9 ± 17.70	32.3 ± 19.25
Ventricular fibrillation	70	75	274 ± 78.54	212 ± 95.24

Data are taken from our paper Adameova et al., 2018 [41]. ND—not detectable. Values for duration of arrhythmias are mean ± S.E.

**Table 2:** Incidence, episodes, onset and duration of arrhythmias due to epinephrine (32 µg/kg) in rats treated with and without a beta-adrenoceptor blocker atenolol (20 mg/kg/day) or an angiotensin receptor blocker, losartan (20 mg/kg/day) treatment for 15 days.

Parameters	Untreated	Atenolol-Treated	Losartan-Treated
Incidence (%)	90	100	90
Episodes (number)	5 ± 1.9	14 ± 4.8 *	8 ± 1.8
Onset (s)	21.5 ± 7.5	24.6 ± 8.8	16.3 ± 2.8
Duration (s)	1.2 ± 0.4	3.4 ± 1.2	2.0 ± 0.9
QT_c_ intervals (s)	0.23 ± 0.007	0.22 ± 0.006	0.21 ± 0.008
QRS interval (s)	0.06 ± 0.002	0.06 ± 0.002	0.06 ± 0.001

Data are taken from our paper Adameova et al., 2019 [43]. Values are mean ± S.E. * *p* < 0.05 vs. untreated.

**Table 3:** Modification of various types of arrhythmias and plasma malondialdehyde (MDA) levels due to epinephrine (32 µg/kg) in rats with and without vitamin E (20 mg/kg/day) or *N*-acetyl-l-cysteine (200 mg/kg/day) treatment for 21 days.

Parameters	Untreated	Vitamin E-Treated	*N*-Acetyl-l-Cysteine-Treated
Onset of arrhythmias (s)	12 ± 1.2	18 ± 1.8 *	20 ± 1.2 *
Duration of arrhythmias (s)	145 ± 5.3	24 ± 1.6 *	22 ± 0.8 *
Singles (number/5 min)	22 ± 2.2	9 ± 0.7 *	10 ± 1.1 *
Salvos (number/5 min)	12 ± 1.0	3 ± 0.5 *	5 ± 0.4 *
Ventricular tachycardia (number/5 min)	45 ± 6.7	22 ± 3.6 *	20 ± 3.2 *
MDA levels before epinephrine (µmol/L)	1.53 ± 0.10	1.25 ± 0.08 *	1.43 ± 0.03
MDA levels after epinephrine (µmol/L)	154 ± 8.21	67 ± 4.90 *	21 ± 2.82 *

Data are taken from our paper Sethi et al., 2009 [15]. Values are mean ± S.E. * *p* < 0.05 vs. untreated.

**Table 4:** Electrocardiographic and oxidative stress parameters in rat hearts 21 days after coronary artery occlusion with or without vitamin E (25 mg/kg/day) treatment.

Parameters	Control	Untreated	Vitamin E—Treated
ST segment (mV)	0.02 ± 0.003	0.11 ± 0.01 *	0.04 ± 0.002 ^#^
QT_c_ interval (ms)	340 ± 20	510 ± 26 *	320 ± 18 ^#^
Q wave appearance (%)	ND	28	4.0
PVC incidence (%)	ND	18	4.0
Conjugated dienes (µmol/mg tissue lipids)	35.8 ± 2.7	62.2 ± 3.1 *	42.5 ± 2.6 ^#^
Malondialdehyde levels (µmol/mg tissue lipids)	3.1 ± 0.2	4.8 ± 0.2 *	2.6 ± 0.1 ^#^

Data are taken from our paper Sethi et al., 2000 [59]. ND—not detectable; values are mean ± S.E. * *p* < 0.05 vs. control; ^#^ *p* < 0.05 vs. untreated.

**Table 5:** Electrocardiographic parameters in rat hearts 21 days after coronary artery occlusion with or without ACE inhibitor, imidapril (1 mg/kg/day) treatment.

Parameters	Control	Untreated	Imidapril-Treated
ST segment (mV)	0.03 ± 0.004	0.13 ± 0.02 *	0.04 ± 0.003 ^#^
QT_c_ interval (ms)	378 ± 14	548 ± 9 *	423 ± 9 ^#^
Q wave appearance (%)	ND	25	25
PVC incidence (%)	ND	14	5
QRS duration (ms)	0.10 ± 0.01	0.09 ± 0.01	0.10 ± 0.01

Data are taken from our paper Ren et al., 1998 [66]. ND—not detectable; values are mean ± S.E. * *p* < 0.05 vs. control; ^#^ *p* < 0.05 vs. untreated.
